# A natural history study of pediatric patients with early onset of GM1 gangliosidosis, GM2 gangliosidoses, or gaucher disease type 2 (RETRIEVE)

**DOI:** 10.1186/s13023-024-03409-1

**Published:** 2024-12-05

**Authors:** Bénédicte Héron, Spyros Batzios, Eugen Mengel, Roberto Giugliani, Marc Patterson, Matthias Gautschi, Peter Cornelisse, Luba Trokan, Barbara Schwierin, Marianne Rohrbach

**Affiliations:** 1 Department of Pediatric Neurology, Reference Centre for Lysosomal Diseases, Armand Trousseau Hospital and Hospitalo-Universitaire Federation I2-D2, AP-HP. Sorbonne-Université, Paris, France; 2 Great Ormond Street Hospital for Children, NHS Foundation Trust, London, UK; 3 SphinCS GmbH, Hochheim, Germany; 4 Department of Genetics UFRGS, Medical Genetics Service HCPA, DR Brasil HCPA, INAGEMP, DASA, and Casa Dos Raros, Porto Alegre, Brazil; 5 Division of Child and Adolescent Neurology, Departments of Neurology, Pediatrics and Medical Genetics, Mayo Clinic Children’s Center, Rochester, MN USA; 6 Department of Paediatrics and Institute of Clinical Chemistry, Swiss Reference Centre for Inborn Errors of Metabolism, Site Berne, University Hospital Bern, Inselspital, Bern, Switzerland; 7 Idorsia Pharmaceuticals Ltd, Allschwil, Switzerland; 8 Division of Metabolism and Children’s Research Center, Reference Center for Inborn Errors of Metabolism, University Children’s Hospital of Zurich, University of Zurich, Zurich, Switzerland

**Keywords:** GM1 gangliosidosis, GM2 gangliosidoses, Gaucher disease type 2, Lysosomal storage disorder, Infants, Natural history

## Abstract

**Background:**

The GM1 and GM2 gangliosidoses and type 2 Gaucher disease (GD2) are inherited lysosomal storage disorders with most cases having symptom onset in infancy and reduced life expectancy. The conditions are rare, and there is therefore a need for accurate and up to date information concerning the disease course and survival to assist in the design of clinical trials. RETRIEVE is a natural history study aiming to: (1) collect data on the survival of patients with early-onset (onset of first neurological manifestation before 24 months of age) GM1, GM2, or GD2; (2) collect data that could constitute a historical control group for future clinical trials; and (3) evaluate whether the conditions can be assessed together in a single interventional clinical trial. Group A included patients who were deceased or with unknown survival status at enrollment and was thus limited to retrospective data. Group B included patients who were alive at enrollment, who were followed prospectively with additional retrospective data collection.

**Results:**

Group A included 185 patients (60 with GM1, 78 with GM2, and 47 with GD2), and Group B included 40 patients (18 with GM1, 16 with GM2, and 6 with GD2). Mean and median age at diagnosis and age at onset of first neurological manifestation were youngest in patients with GD2 and oldest in patients with GM2 in both groups. In Group A, median (95% CI) survival was 19.0 (18.0, 22.0), 44.0 (37.0, 51.9) and 14.0 (10.0, 16.0) months in patients with GM1, GM2 and GD2, respectively. In Group B, hypotonia was experienced by most patients with GM1 (17/18, 94.4%), and was less common in patients with GM2 (12/16, 75.0%) and GD2 (4/6, 66.7%). Strabismus and splenomegaly were reported in all six patients with GD2.

**Conclusions:**

RETRIEVE is one of the largest natural history studies of GM1, GM2, and GD2. Results were generally consistent with the published literature, with differences potentially due to variation in inclusion criteria. The difference in median survival between patients with early-onset GM1, GM2, and GD2 reported in this study suggests that the three diseases should not be pooled for study in clinical trials.

**Supplementary Information:**

The online version contains supplementary material available at 10.1186/s13023-024-03409-1.

## Background

The gangliosidoses are a heterogeneous group of inherited lysosomal storage disorders, in which pathogenic biallelic mutations in genes encoding lysosomal hydrolases result in accumulation of GM1 or GM2 gangliosides [1–3]. Depending on the gene that is mutated, GM2 gangliosidoses present in three main forms: Tay Sachs disease, Sandhoff disease, and AB variant (also known as GM2-activator-deficiency) [4]. Symptoms of GM1 and GM2 gangliosidoses may become apparent during infancy or childhood and lead to death in the early years of life [2, 3]. However, there is no consensus on the age of first symptom for early-infantile/late-infantile/juvenile GM1 and GM2 [2–7]. Gaucher disease (GD), another inherited lysosomal storage disorder, is considered one of the most common sphingolipidoses [8]. Gaucher disease presents as a phenotypic spectrum, historically classified into three major types – type 1, type 2, and type 3. Of these, type 2 GD (GD2) is characterized by severe and progressive neurological impairment in the first months of life [8]. Common to GM1, GM2, and GD2 are the neurodegenerative course, early mortality [2, 3, 8], and the underlying pathology that involves accumulation of complex glycosphingolipids and leads to their classification as lysosomal storage diseases [1, 5].

With only a few, small studies published, there is a lack of accurate and up-to-date information on the natural course and survival of the three conditions. Furthermore, there is currently no effective therapy, nor any approved disease-modifying treatment, for GM1, GM2, or GD2. Due to difficulties designing and conducting randomized clinical trials assessing interventions for these very rare diseases, observational studies could provide reference information and serve as a control group for clinical studies [9]. Owing to the similarities in the neurological manifestations, biochemical pathway (sphingolipid degradation), early onset, and short lifespan of patients with the three conditions, it may be possible to investigate them together in one clinical study. Anyway, the specific data for each of the 3 diseases are well identified and provide a disease-specific information.

The objectives of the RETRIEVE study were, therefore, to: (1) collect data on survival of pediatric patients with early-onset (onset of first neurological manifestation before 24 months of age) GM1, GM2, or GD2; (2) collect data that could constitute a historical control group for future clinical trials; and (3) evaluate whether the three conditions can be assessed together in a single interventional clinical trial.

## Methods

### Study design

RETRIEVE is an international, multi-center, observational study of patients with GM1, GM2, or GD2, with two groups: Group A included patients who were deceased or whose survival status was not known at enrollment, while Group B included patients who were alive at enrollment. Group B was followed prospectively in addition to retrospective data collection.

The study was conducted in compliance with Good Epidemiology Practice guidelines [10, 11], the ethical principles arising from the Declaration of Helsinki revised in 2013, and all current local regulations. Ethics Committee/Institutional Review Board approval was obtained as required by applicable site policies, national privacy regulations, and other state and local laws relating to medical information. Informed consent of the parent or legal guardian was obtained as required by local law. The study has been registered at ClinicalTrials.gov, NCT04470713.

### Setting and participants

RETRIEVE was conducted in hospitals/clinical centers managing pediatric patients with GM1, GM2, or GD2 in 17 sites in Belgium, Brazil, France, Germany, Italy, Portugal, Spain, Switzerland, UK, and the USA (Additional file 1: Table S1). For Group A, retrospective data were collected between 23 July 2019 and 15 June 2021, and for Group B, data were collected from 13 November 2019 until the end of study (31 October 2021).

Inclusion criteria included a diagnosis of GM1, GM2 (Tay-Sachs, Sandhoff, AB variant), or GD2 confirmed by biochemical (enzyme activity) or genetic testing, or both; date of birth on or after 1 January 2000; onset of the first neurological manifestation before 24 months of age; and informed consent of a parent or legal guardian.

### Variables

Data collected for this study included variables assessed or measured in the course of standard clinical care. For Groups A and B, this included disease of interest, date of birth, sex, race, ethnicity, diagnosis (including date, gene mutations, and enzyme activity test results as applicable), date of onset of first neurological manifestation, date of insertion of G-tube or other permanent enteral support, start and stop dates of any investigational/off-label treatment (defined as hematopoietic stem cell transplant, miglustat [with or without ketogenic diet], and other investigational treatments), and occurrence of death (yes/no), date of death, or date of last contact alive if survival status was unknown.

Additional data collected for Group B included date of first occurrence of signs/symptoms (e.g., dyskinesia, swallowing problems), dates of gain and loss of abilities in the gross and fine motor domains and cognitive/social domain, start and stop date of pneumonia and seizures, type of seizures, start date of pulmonary physiotherapy, date of tracheostomy, presence of hepatomegaly or splenomegaly, and start and stop date of any treatment indicated for major signs/symptoms. For patients with GD2, results from blood tests (red blood cells, hemoglobin, and thrombocytes) were also collected.

For patients whose survival status was unknown at study enrollment, the last date that the patient was known to be alive was used to compute the patient’s age.

### Data sources and management

All data for Group A were collected retrospectively. For Group B, data collection was retrospective for the time from birth to enrollment, and prospective from enrollment to the end of the study. For both groups, retrospective data were collected from existing data sources such as physician notes, medical charts, and discharge letters. For Group B, prospective data were collected from standard routine visits.

No assessments or evaluations outside of routine practice were required, and there was no visit schedule apart from the enrollment visit for Group B patients. Investigators and site personnel entered data into an electronic data capture system via a secure network with secure access features. Source data verification was performed by clinical research associates.

### Study size and *bias*

This observational, natural history study did not test hypotheses, and therefore no formal sample size calculations were performed; rather, the study size was set to be as large as possible to allow sufficiently accurate estimation of parameters and is limited by the availability of patients with those rare diseases. It was assumed that at least 150 patients in Group A (at least 60 patients with GM1, 80 patients with GM2, and 10 patients with GD2), and at least 30 patients alive at time of enrollment (Group B) would provide sufficient sample size overall and in each disease. To minimize selection bias, centers were asked to include all eligible patients from their center in the study.

### Statistical methods

All statistical analyses were descriptive in nature and were performed on the full analysis set, which comprised all patients who met study inclusion criteria and for whom data had been entered into the electronic data capture system. Patients known to be deceased but without a recorded date of death were excluded from analyses requiring a date of death.

Overall survival, defined as time from birth to death, was analyzed using Kaplan–Meier methods to estimate median survival time and corresponding 95% CI for each disease group (GM1, GM2, and GD2). If a patient’s status was alive or unknown, then the date of last contact alive was used to calculate the survival time.

Subgroup analyses of overall survival were performed by age at onset of first neurological manifestation (0–6, 0–12, and > 24 months). No imputation for missing data was performed.

All other variables were analyzed descriptively using appropriate summary statistics.

## Results

### Participants

**Fig. 1:**
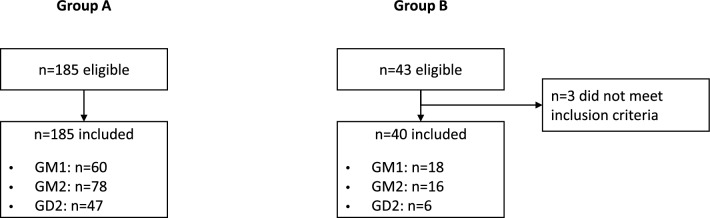
Patient flow diagram

A total of 225 patients were included by the participant centres (Additional file 1 : Table S2). For Group A, 185 patients were included; 60 had been diagnosed with GM1, 78 with GM2, and 47 with GD2 (Fig. 1). Of those diagnosed with GM2, 48 (61.5%) had Tay-Sachs, 29 (37.2%) had Sandhoff disease, and one patient had AB variant. For Group B, 43 patients were initially deemed eligible but three did not meet inclusion criteria (onset of first neurological manifestation before 24 months of age) and were not included in the full analysis set. Of the remaining 40 patients, 18 had been diagnosed with GM1, 16 with GM2, and 6 with GD2 (Fig. 1). Of those diagnosed with GM2, seven patients had Tay-Sachs, eight patients had Sandhoff disease, and one patient had the AB variant. Median follow-up for patients in Group B was 10.3 months (range, 0.7 to 23.2 months).

Demographic characteristics for both groups are shown in Table 1. The birth decade of patients in Group A was roughly balanced between 2000–2010 and 2011–2021, whereas in Group B 82.5% of the patients were born in 2011–2021 (three patients with GM1 and four with GM2 were born in 2000–2010). For both groups, patients with GM2 had the highest mean age at enrollment and those with GD2 had the youngest mean age at enrollment. In Group A, age at enrollment was not calculated for seven patients with unknown survival status at enrollment, and for three patients for whom a date of death was missing.

**Table 1:** Patient baseline demographic characteristics

Group A	GM1 n = 60	GM2 n = 78	GD2 n = 47	Total n = 185
Female, n (%)	39 (65.0)	45 (57.7)	25 (53.2)	109 (58.9)
Birth decade				
2000–2010	26 (43.3)	43 (55.1)	26 (55.3)	95 (51.4)
2011–2021	34 (56.7)	35 (44.9)	21 (44.7)	90 (48.6)
Survival status at enrollment, n (%)				
Unknown	4 (6.7)	2 (2.6)	1 (2.1)	7 (3.8)
Deceased	56 (93.3)	76 (97.4)	46 (97.9)	178 (96.2)
Age at enrollment (months), mean ± SD	30.1 ± 36.0	54.2 ± 34.0	17.5 ± 16.2	37.1 ± 34.7
Missing	4	4	2	10

Group B	GM1 n = 18	GM2 n = 16	GD2 n = 6	Total n = 40
Female, n (%)	12 (66.7)	5 (31.3)	3 (50.0)	20 (50.0)
Birth decade				
2000–2010	3 (16.7)	4 (25.0)	0	7 (17.5)
2011–2021	15 (83.3)	12 (75.0)	6 (100.0)	33 (82.5)
Age at enrollment (months), mean ± SD	58.7 ± 63.3	67.8 ± 53.0	19.2 ± 11.5	56.4 ± 55.8

Age at enrollment for deceased patients is equal to age at death. GD2, Gaucher disease type 2

Race and ethnicity were not reported because their collection was not permitted as per local legislation/regulation (in more than one-third of patients), or they were unknown.

### Onset of neurological manifestations and diagnosis

Mean and median age at diagnosis were youngest in patients with GD2 and oldest in patients with GM2 in both groups (Table 2). In both groups, age at onset of first neurological manifestation was youngest for patients with GD2 and oldest for patients with GM2. The delay between first manifestation and diagnosis was longest for GM2, at around a year in Group A and more than 2 years in Group B.

**Table 2:** Disease characteristics

Group A	GM1 n = 60	GM2 n = 78	GD2 n = 47
Age at onset of first neurological manifestation (months)			
Mean ± SD	6.0 ± 5.1	7.8 ± 5.7	4.6 ± 4.2
Median (IQR)	5 (3–8)	8 (3–11)	4 (1–7)
Age at diagnosis (months)			
Mean ± SD	10.9 ± 9.0	19.0 ± 20.1*	9.5 ± 11.4
Median (IQR)	10 (6–14)	14 (11–17) *	8 (3–12)
Time from onset of first neurological manifestation to diagnosis (months)			
Mean ± SD	5.3 ± 8.3	11.5 ± 18.2*	5.1 ± 10.3
Median (IQR)	4 (1–9)	8 (4–13) *	4 (0–7)

Group B	GM1 n = 18	GM2 n = 16	GD2 n = 6
Age at onset of first neurological manifestation (months)			
Mean ± SD	9.4 ± 6.5	9.9 ± 6.8	6.0 ± 5.5
Median (IQR)	9 (4–13)	11 (5–16)	6 (2–8)
Age at diagnosis (months)			
Mean ± SD	20.1 ± 13.2	35.8 ± 27.4	7.5 ± 6.6
Median (IQR)	19 (9–30)	27 (16–49)	8 (1–10)
Time from onset of first neurological manifestation to diagnosis (months)			
Mean ± SD	11.1 ± 8.8	26.2 ± 26.2	1.5 ± 3.6
Median (IQR)	8 (5–19)	14 (6–42)	1 (-1–3)

*Data not available for one patient. GD2, Gaucher disease type 2; IQR, interquartile range; SD, standard deviation

### Survival analysis

#### Group A

**Fig. 2:**
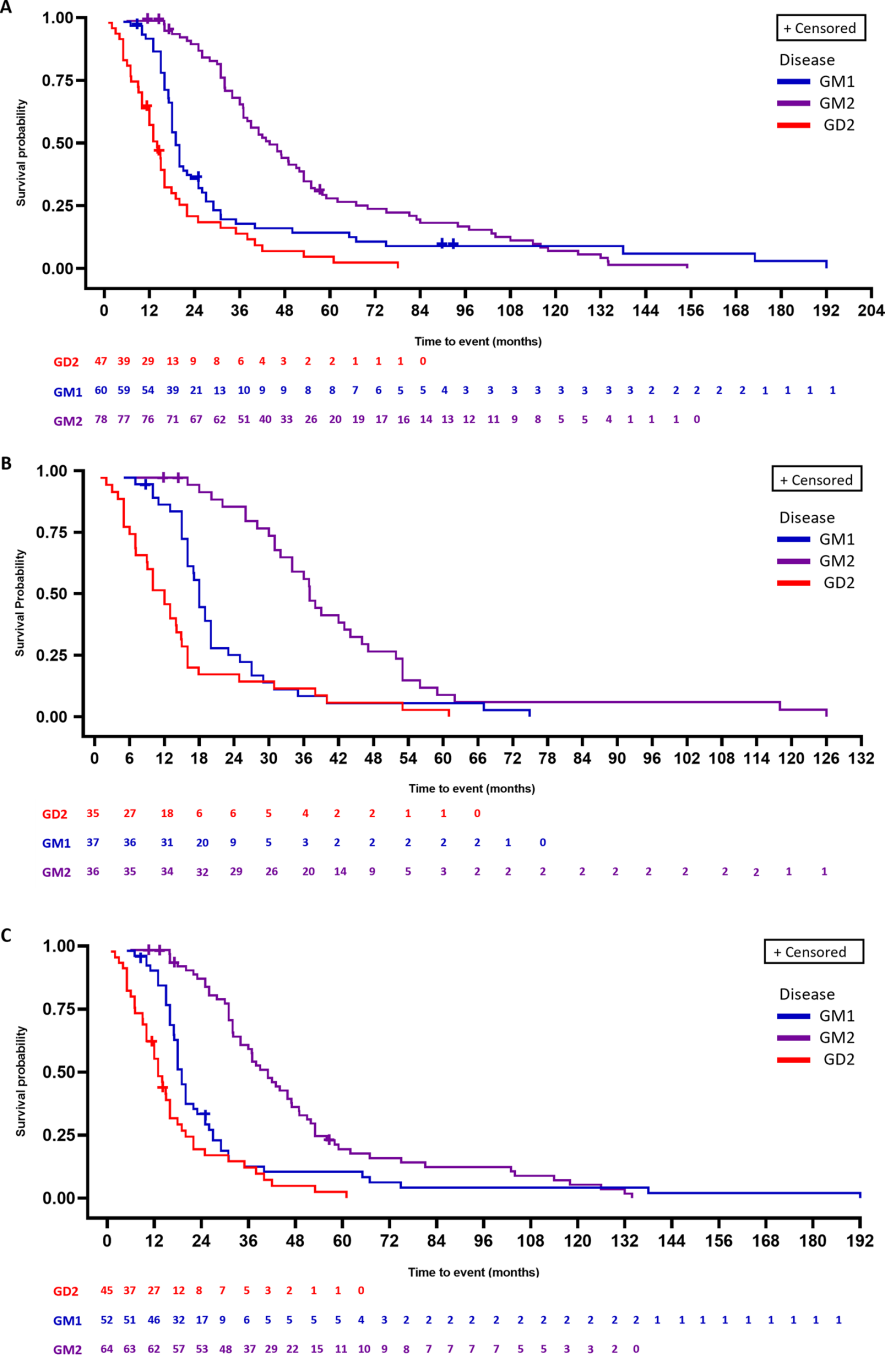
Kaplan–Meier plot of time to death by disease in Group A. **A** Full analysis set (age at onset of neurological manifestations 0-–24 months); **B** age at onset of neurological manifestations 0–6 months; **C** age at onset of neurological manifestations 0–12 months. GD2, Gaucher disease type 2

Survival in Group A is shown in Fig. 2. Median (95% confidence interval [CI]) survival was 19.0 (18.0, 22.0), 44.0 (37.0, 51.9), and 14.0 (10.0, 16.0) months in patients with GM1, GM2, and GD2, respectively (Table 3). Regardless of the width of the time window for age at onset of neurological manifestations (0–6, 0–12, or 0–24 months), median survival was longest in patients with GM2 and shortest in patients with GD2.

**Table 3:** Median survival by age of neurological onset in Group A

Onset of neurological manifestations	GM1 n = 60	GM2 n = 78	GD2 n = 47
0–6 months			
n	37	36	35
Median survival, months (95% CI)	18.0 (16.0, 20.0)	37.0 (31.0, 44.0)	12.0 (7.1, 14.1)
0–12 months			
n	52	64	45
Median survival, months (95% CI)	19.0 (17.1, 22.0)	41.0 (34.0, 47.0)	13.0 (10.0, 16.0)
0–24 months			
n	60	78	47
Median survival, months (95% CI)	19.0 (18.0, 22.0)	44.0 (37.0, 51.9)	14.0 (10.0, 16.0)

CI, confidence interval; GD, Gaucher disease type 2

The primary cause of death in Group A was disease progression (83.9%, 77.6%, and 84.8% of patients with GM1, GM2, and GD2, respectively).

#### Group B

The number of deaths was low in Group B (three in patients with GM1, one with GM2, and one with GD2), so median survival was not calculated. Causes of death in patients with GM1 were pneumonia, lung disorder, and respiratory insufficiency; in GM2 was pneumonia; and in GD2 was disease progression (not further specified).

### Manifestations in Group B

Manifestations reported for patients in Group B are shown in Table 4. Hypotonia was experienced by nearly all patients with GM1 (n = 17/18, 94.4%), and was less commonly observed in patients with GM2 (n = 12/16, 75.0%) and GD2 (n = 4/6, 66.7%). Strabismus and splenomegaly were each reported in all six patients with GD2. The manifestations with earliest mean age of onset in patients with GM1 were dyskinesia (12.0 months), splenomegaly (12.8 ± 4.6 months), and swallowing problems (13.8 ± 12.2 months), although patient numbers were small. Swallowing problems (mean age 5.4 ± 6.5 months) and splenomegaly (5.8 ± 6.9 months) were also among the manifestations observed earliest in patients with GD2, along with strabismus (mean 5.3 [SD, 8.6] months). For patients with GM2, the only manifestation with mean age of first onset younger than 18 months was hypotonia (13.3 ± 9.0 months).

**Table 4:** Manifestations in Group B

	GM1 n = 18	GM2 n = 16	GD2 n = 6	Total n = 40
Hypotonia				
n (%)	17 (94.4)	12 (75.0)	4 (66.7)	33 (82.5)
Age at onset (months), mean ± SD (n)	17.3 ± 16.3 (12)	13.3 ± 9.0 (11)	10.0 ± 4.2 (4)	14.6 ± 12.4 (27)
Hypertonia				
n (%)	11 (61.1)	7 (43.8)	5 (83.3)	23 (57.5)
Age at onset (months), mean ± SD (n)	21.5 ± 16.4 (8)	46.8 ± 45.5 (5)	11.3 ± 4.0 (4)	26.5 ± 28.9 (17)
Spasticity				
n (%)	10 (55.6)	11 (68.8)	3 (50.0)	24 (60.0)
Age at onset (months), mean ± SD (n)	17.6 ± 14.1 (8)	35.0 ± 20.2 (11)	9.0 (1)	26.8 ± 19.5 (20)
Strabismus				
n (%)	7 (38.9)	3 (18.8)	6 (100)	16 (40.0)
Age at onset (months), mean ± SD (n)	16.3 ± 9.7 (4)	54.7 ± 68.8 (3)	5.3 ± 8.6 (4)	22.7 ± 38.0 (11)
Nystagmus				
n (%)	6 (33.3)	4 (25.0)	0	10 (25.0)
Age at onset (months), mean ± SD (n)	33.2 ± 40.7 (5)	39.0 ± 35.6 (4)	0	35.8 ± 36.3 (9)
Diminished eyesight				
n (%)	8 (44.4)	10 (62.5)	1 (16.7)	19 (47.5)
Age at onset (months), mean ± SD (n)	20.0 ± 21.2 (5)	32.3 ± 31.5 (9)	0	27.9 ± 28.0 (14)
No eyesight				
n (%)	4 (22.2)	4 (25.0)	0	8 (20.0)
Age at onset (months), mean ± SD (n)	24.7 ± 18.6 (3)	30.7 ± 2.5 (3)	0	27.7 ± 12.3 (6)
Diminished hearing				
n (%)	5 (27.8)	2 (12.5)	1 (16.7)	8 (20.0)
Age at onset (months), mean ± SD (n)	17.0 ± 13.1 (3)	43.0 (1)	0	23.5 ± 16.8 (4)
No hearing				
n (%)	1 (5.6)	1 (6.3)	0	2 (5.0)
Age at onset (months), mean ± SD (n)	18.0 (1)	19.0 (1)	0	18.5 ± 0.7 (2)
Ataxia				
n (%)	4 (22.2)	7 (43.8)	0	11 (27.5)
Age at onset (months), mean ± SD (n)	15.0 (1)	49.0 ± 32.0 (6)	0	44.1 ± 31.9 (7)
Dyskinesia				
n (%)	1 (5.6)	3 (18.8)	2 (33.3)	6 (15.0)
Age at onset (months), mean ± SD (n)	12.0 (1)	73.7 ± 36.6 (3)	18.0 (1)	50.2 ± 41.3 (5)
Swallowing problems				
n (%)	14 (77.8)	12 (75.0)	5 (83.3)	31 (77.5)
Age at onset (months), mean ± SD (n)	13.8 ± 12.2 (10)	48.7 ± 43.8 (11)	5.4 ± 6.5 (5)	27.0 ± 34.6 (26)
Pneumonia				
n (%)	7 (38.9)	9 (56.3)	3 (50.0)	19 (47.5)
Age at onset (months), mean ± SD (n)	18.4 ± 11.3 (5)	63.1 ± 49.0 (9)	13.7 ± 4.2 (3)	41.2 ± 42.5 (17)
Seizures				
n (%)	14 (77.8)	13 (81.3)	4 (66.7)	31 (77.5)
Age at onset (months), mean ± SD (n)	32.7 ± 26.7 (12)	30.9 ± 18.4 (12)	12.8 ± 7.4 (4)	29.1 ± 21.9 (28)
Hepatomegaly				
n (%)	9 (50.0)	2 (12.5)	5 (83.3)	16 (40.0)
Age at onset (months), mean ± SD (n)	32.1 ± 56.1 (9)	39.0 ± 18.4 (2)	6.6 ± 7.5 (5)	25.0 ± 43.4 (16)
Splenomegaly				
n (%)	4 (22.2)	1 (6.3)	6 (100)	11 (27.5)
Age at onset (months), mean ± SD (n)	12.8 ± 4.6 (4)	52.0 (1)	5.8 ± 6.9 (6)	12.5 ± 14.6 (11)

GD2, Gaucher disease type 2; SD, standard deviation. “Ataxia” was not defined further. All variables were assessed or measured during standard clinical care for the patients

### Treatments

All patients in Group B received at least one pharmacological treatment for major manifestations of their condition; the most common treatments were levetiracetam (n = 18, 45% of patients) and diazepam (n = 12, 30% of patients). Investigational or off-label treatment was more common in Group B than Group A (37.5% versus 10.3% of patients, respectively).

In Group B, the most frequently administered non-pharmacological treatments in patients with GM1 and GM2 were physiotherapy (16.7% and 56.3% of patients) and speech rehabilitation (11.1% and 43.8% of patients). No patients with GD2 received non-pharmacological treatment. Also in Group B, no patients with GM1, one patient with GM2 and two patients with GD2 had a tracheostomy. In Group A, permanent enteral support was received by 60.0%, 71.8%, and 55.3% of patients with GM1, GM2, and GD2, respectively, at a mean (SD) age of 17.0 (12.2), 29.6 (16.8), and 16.6 (13.6) months, respectively. In Group B, 61.1%, 68.8%, and 83.3% of patients with GM1, GM2, and GD2, respectively, received permanent enteral support, at a mean (SD) age of 38.4 (36.7), 41.3 (37.8), and 10.4 (5.9) months, respectively.

### Functional abilities in Group B

**Fig. 3:**
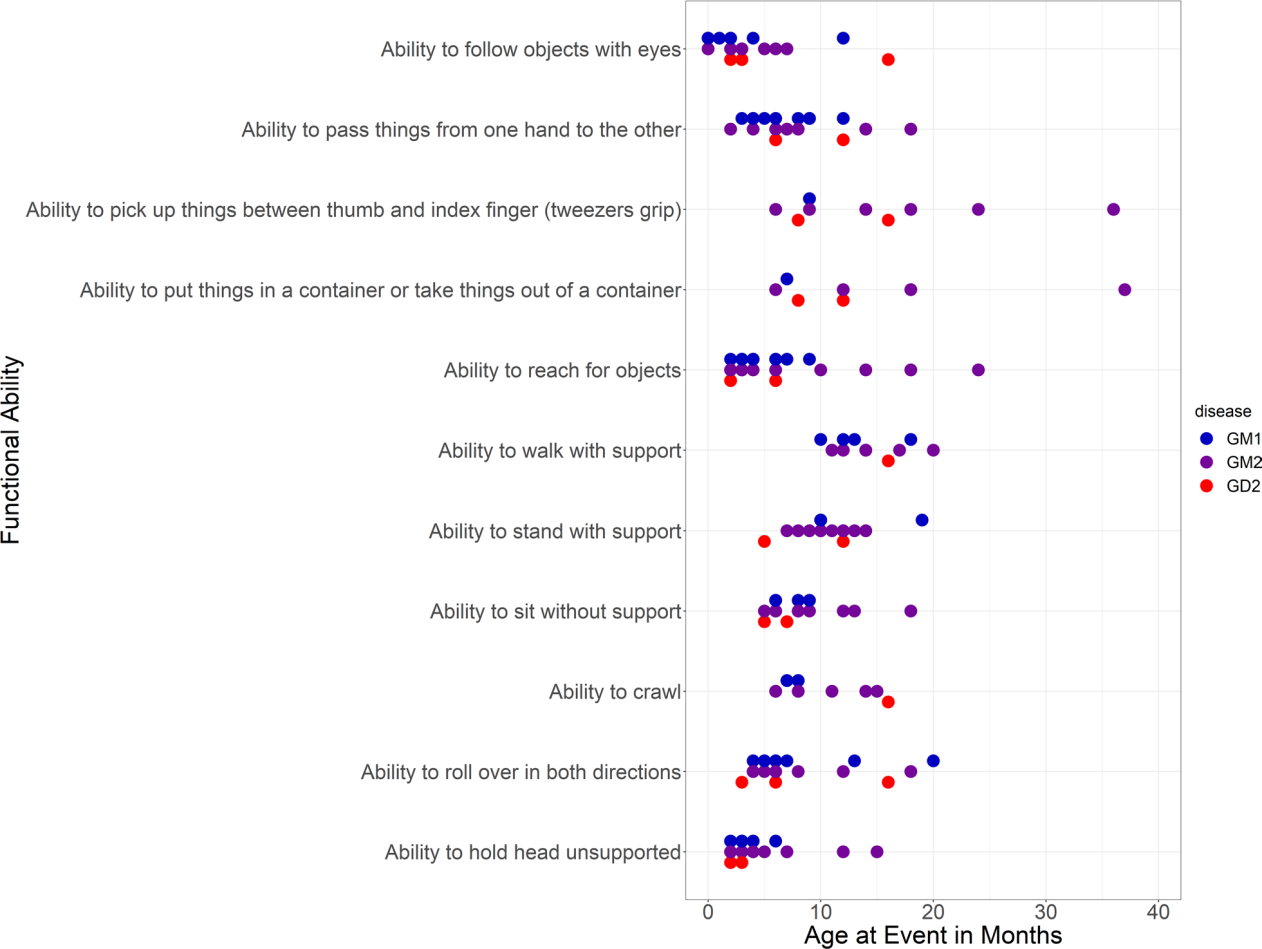
Age at loss of functional abilities in Group B. Two outliers with an age of 129 months in the categories ‘ability to stand with support’ and ‘ability to walk with support’ are not shown. Data were not available for all patients; dots for different abilities do not necessarily represent the same patients

Age at loss of functional abilities in Group B is shown in Fig. 3. For certain patients, it was unknown whether a particular functional ability was gained and/or lost; for some patients, it was known that a specific ability was lost, but the date of the loss was unknown. Patients with onset of neurological manifestations after 12 months were more likely to gain the abilities to sit without support, stand with support, and walk with support than those with onset ≤ 12 months, but with small patient numbers this should be interpreted with caution.

## Discussion

RETRIEVE collected retrospective information on 60, 78, and 47 patients with early-onset GM1, GM2, or GD2, respectively, over 20 years (Group A), and prospective data on 40 patients who were alive at enrollment (Group B), making it one of the largest natural history studies in this area. Group B represents patients who may be recruited into clinical studies, and these results therefore provide important information for those planning clinical trials.

Results of the survival analysis of Group A (with retrospective data collection) were generally consistent with those in the literature, although inclusion criteria differ between studies. Median survival for patients with GM1 in Group A was 19.0 (95% CI, 18.0, 22.0) months, similar to that reported by Lang et al. describing 154 patients with manifestation onset before 12 months (average age at death, 18.9 months) [6]. However, this was shorter than that reported by Utz et al. for eight patients (median survival 45.91 months), perhaps due to the small patient number in that analysis [2].

For patients with GM2, the median survival in Group A in RETRIEVE of 44.0 (95% CI, 37.0, 51.9) months is very similar to that reported by Utz et al. (median survival 43.43 months for 15 patients) [2] and slightly longer than that reported by Smith et al. (mean age at death 36.3 months for 55 patients) [7]; both of these studies included patients with manifestation onset ≤ 12 months. When considering the 64 patients with GM2 in Group A with age of neurological onset ≤ 12 months, the median survival remains similar to that reported in the literature (41.0 months; 95% CI, 34.0, 47.0).

In patients with GD2, the median survival in RETRIEVE of 14.0 (95% CI, 10.0, 16.0) months is in line with the average age of death of 11.7 months reported by Mignot et al. (76 patients) [12] and mean age at death of 19.2 months reported by Lal et al. (23 patients) [13].

The wide range in median survival between the three diseases in RETRIEVE (44 months in GM2 versus 14 months in GD2 and 19 months in GM1) suggest that the diseases should not be pooled for study in clinical trials. However, median survival for the individual diseases was broadly consistent with the literature, and broadly consistent across different age of onset ranges (0–6, 0–12, and 0–24 months). This could indicate that patients with age of onset ≤ 24 months have a similar disease course to those with earlier age of onset and could therefore be grouped in interventional trials. Small patient numbers in each subgroup in RETRIEVE preclude analysis of whether these patients had a different prognosis or age of death.

Age at first onset of manifestation for patients with GM1 in RETRIEVE was later than has been reported in the literature: 6.0 ± 5.1 months and 9.4 ± 6.5 months for Groups A and B in RETRIEVE, respectively, versus 2.8 months reported by Lang et al. [6] and 1.5 months reported by Utz et al. [2]. This likely reflects the differing inclusion criteria in the studies; varying age of onset for early and late infantile GM1 and GM2 have been used in the literature. For (early) infantile (Type 1) GM1, the age at first manifestation has been described as birth to 6 months [14] and before 12 months [3, 6] and for late infantile GM1 between 7 months and 3 years, [14] 1–3 years, [3] and around 1 year [5]. For GM2, the age at first manifestation has been variously reported as during the first year of life, [5] 3–6 months, [5] 5 months [4], and ≤ 12 months [7]. To generate data outside of the classically defined infantile diseases, RETRIEVE enrolled patients with age of first manifestation up to 24 months.

This study reports manifestations experienced by patients in Group B, which were consistent with the literature. Hypotonia was the most common manifestation experienced by patients with GM1 in Group B (94.4%), similar to the 96–100% reported in infantile-onset GM1 in the literature [2, 6, 14]. Three-quarters of patients with GM2 in Group B had hypotonia, consistent with reports of 68% and 96% in previous studies [4, 7]. Strabismus and splenomegaly were reported in all six patients with GD2 in Group B; Mignot et al. report 9/15 patients had strabismus and 14/15 had splenomegaly [12], and Lal et al. report 18/23 patients had strabismus [13].

In patients with GM2, the mean age at diagnosis in Group B (35.8 ± 27.4 months) is later than in Group A (19.0 ± 20.1 months). Age at diagnosis in Group B is also later than in reports in the literature. Similar to survival, the differences to the literature may be due to differing inclusion criteria (age at first manifestation ≤ 12 months [7] or not defined [2, 4]); another potential explanation may be the methods used for diagnosis [2, 4, 7]. Patients with GM2 also experienced a long delay between appearance of first manifestation and diagnosis, of more than 2 years in Group B. For patients with GM1 the diagnostic delay was shorter at around 1 year in Group B; the difference may be due to the slower progression seen with GM2 versus GM1.

The strengths of this natural history study include the relatively large number of patients, and the relevance of the results for prospective clinical trials. The median survival analysis of Group A informs whether patients with different diseases and different age of onset could be pooled together in clinical studies, while the data collected for Group B could form an external group for future trials.

This study also has some limitations. The short observation time and small patient number for Group B limits conclusions that can be drawn from the data. In addition, in Group B data were collected both retrospectively and prospectively (i.e., data collected before and after enrollment in the study). For instance, dates of gain and loss of functional abilities that occurred before study enrollment were collected retrospectively, and therefore may be less precise than those collected post-enrollment, when the physician was aware of data collection. Additionally, no standardized tools were used for the assessment of functional abilities, and age at gain of function was not always recorded.

## Conclusions

The data collected in RETRIEVE provide important information for future clinical trials in these rare diseases. Analysis of Group A showed that patients had broadly similar overall survival independent of age of first manifestation (0–6, 0–12, and 0–24 months), and consistent with the literature, suggesting that patients with different age of onset have a similar disease course and could be pooled in clinical trials. However, the wide range in survival between the disease groups (GM1, GM2, and GD2) indicate that patients with these conditions should not be analyzed together in clinical studies. Data collected prospectively in Group B could form the basis for a historical control group in future trials.

## Supplementary Information

**Additional file 1. Table S1.** Participating Centres. **Table S2.** Number of patients recruted per Centre and per Group for each disease.

## Data Availability

In addition to Idorsia Pharmaceuticals Ltd’s existing clinical trial disclosure activities, the company is committed to implementing the Principles for Responsible Clinical Trial Data Sharing jointly issued by the European Federation of Pharmaceutical Industries and Associations (EFPIA) and the Pharmaceutical Research and Manufacturers of America (PhRMA). Requests for data sharing, of any level, can be directed to clinical-trials-disclosure@idorsia.com for medical and scientific evaluation.

## Abbreviations

CI: Confidence interval
GD2: Gaucher disease type 2
