# Supplementary material for: A natural history study of pediatric patients with early onset of GM1 gangliosidosis, GM2 gangliosidoses, or gaucher disease type 2 (RETRIEVE)
Source: Orphanet J Rare Dis. 2024 Dec 5;19:459. doi: 10.1186/s13023-024-03409-1 (PMC11619657; doi:10.1186/s13023-024-03409-1)
Supplement: Supplementary file 1 — Additional file 1. Table S1. Participating Centres. Table S2. Number of patients recruted per Centre and per Group for each disease. [file 13023_2024_3409_MOESM1_ESM.docx]

# Supplementary table S1: Participating Centres

| 1001 | Etienne Sokal, MD, PHD | Paediatric Clinical Investigation Center, Cliniques Universitaires St Luc, Brussels, Belgium |
| --- | --- | --- |
| 1101 | Spyros Batzios, MD, MSc, PhD | Great Ormond Street Hospital for Children NHS Foundation Trust, London, UK |
| 1102 | Suresh Vijay, MBBS FRCPCH | Birmingham Women’s and Children’s NHS Foundation Trust, Birmingham, UK |
| 1201 | Joel Charrow, MD, Professor of Pediatrics | Division of Genetics, Birth Defects and Metabolism, Ann and Robert H. Lurie Children’s Hospital of Chicago, Chicago, Illinois, US |
| 1203 | Ozlem Goker-Alpan, MD | Lysosomal & Rare Disorders Research & Treatment Center (LDRTC)/ Fairfax, Virginia, US |
| 1205 | Marc C. Patterson, MD, FRACP, Professor of Neurology, Pediatrics and Medical Genetics | Mayo Clinic College of Science and Medicine, Rochester, Minnesota, US |
| 2001 | Roberto Giugliani, MD PhD | Department of Genetics UFRGS, Porto Alegre, Brazil |
| 3002 | Bénédicte Héron, MD | AP-HP, Hôpitaux Universitaires Est Parisien, Hôpital Trousseau Reference Centre for Lysosomal Diseases, Paris, France |
| 4003 | Eugen Mengel, MD | SphinCS GmbH, Hochheim, Germany |
| 5001 | Isabella Moroni, MD | Department of Paediatric Neurosciences, IRCCS Foundation, Carlo Besta Neurological Institute, Milan, Italy |
| 5002 | Elena Procopio, MD | Department of Metabolic Diseases, Meyer University Hospital, Florence, Italy |
| 7001 | Ana Gaspar, MD | Metabolic Diseases Unit - Pediatric Department, Centro Hospitalar Universitário Lisboa Norte, Lisboa, Portugal |
| 7002 | Elisa Leão Teles, MD | Reference Centre of Inherited Metabolic Diseases, Centro Hospitalar Universitário de São João, Porto, Portugal |
| 8001 | Pilar Giraldo MD PhD | Hospital QuironSalud Zaragoza, Spain |
| 8002 | María del Mar O´Callaghan Gordo, MD, PhD | Neurometabolic Department, Hospital Sant Joan de Déu, Barcelona, Spain |
| 9001 | Matthias Gautschi, MD PhD | Department of Paediatrics and Institute of Clinical Chemistry, University Hospital Bern, Inselspital, Bern, Switzerland |
| 9002 | Marianne Rohrbach, MD PhD | Divison of metabolism and childrens‘ research center, Universitäts-Kinderspital, Zuerich, Switzerland |

# Supplementary table S2: number of patients recruited per centre and per Group for each disease

|  | Group A GD2 | Group A GM1 | Group A GM2 | Group B GD2 | Group B GM1 | Group B GM2 |
| --- | --- | --- | --- | --- | --- | --- |
| 1001 | 1 | 0 | 2 | 0 | 0 | 0 |
| 1101 | 2 | 5 | 3 | 0 | 0 | 1 |
| 1102 | 4 | 5 | 8 | 0 | 1 | 0 |
| 1201 | 1 | 6 | 3 | 0 | 2 | 0 |
| 1203 | 3 | 0 | 0 | 5 | 0 | 0 |
| 1204 | 0 | 0 | 0 | 0 | 0 | 0 |
| 1205 | 0 | 4 | 3 | 0 | 0 | 1 |
| 2001 | 3 | 4 | 0 | 0 | 3 | 0 |
| 3002 | 15 | 20 | 33 | 0 | 8 | 4 |
| 4003 | 4 | 2 | 6 | 0 | 2 | 8 |
| 5001 | 3 | 0 | 3 | 0 | 1 | 1 |
| 5002 | 2 | 6 | 3 | 0 | 0 | 0 |
| 7001 | 2 | 4 | 2 | 0 | 0 | 0 |
| 7002 | 0 | 0 | 3 | 0 | 0 | 0 |
| 8001 | 4 | 0 | 0 | 1 | 0 | 0 |
| 8002 | 1 | 3 | 5 | 0 | 1 | 0 |
| 9001 | 2 | 0 | 0 | 0 | 0 | 1 |
| 9002 | 0 | 1 | 4 | 0 | 0 | 0 |
| Total | 47 | 60 | 78 | 6 | 18 | 16 |
